# II—A Grave National Deficiency

**Published:** 1917-08-18

**Authors:** R. Fortescue Fox

**Affiliations:** Hon. Medical Director of the Red Cross Clinic in London for the Physical Treatment of Disabled Officers.


### PHYSICAL ORTHOPÆDICS.

### II.—A Grave National Deficiency.

By R. FORTESCUE FOX, M.D. (Hon. Medical Director of the Red Cross Clinic in London for the Physical Treatment of Disabled Officers).

The Red Cross Clinic for the physical treatment of disabled officers was opened in London exactly a year ago, in the hope that it might serve as an object-lesson in these unfamiliar forms of physical treatment. This is a small clinic, dealing with fifty or sixty officers daily, with a special system of measurements and records. We have found, as others have found who are in charge of physical treatment, whether in England or France, that the best results are obtained in early cases. Cases of hopeless wasting and anchylosis have often been sent for treatment, but I am glad to say that many cases of wound or fracture are now submitted to hydro-massage and manipulation at an early stage, with excellent results. It is often quite unnecessary to wait for the fracture to be united or for the wound to heal.

Perhaps the largest and best-equipped hydrological department in the British Islands is to be opened in the present month, at the Orthopædic Hospital at Shepherd's Bush. This is designed to treat 500 cases per day by all forms of remedial baths, and will have a complete system of measurements and records. Similar departments have been already opened at the new orthopædic centres, including those at Bellahoustoun near Glasgow and at Bangour in Midlothian, and, I believe, also in Aberdeen.

Local whirlpool baths for the arm or leg have also been set up at many civil hospitals where wounded soldiers are under treatment, and at the Red Cross hospitals at Netley, Brighton, Sidmouth and elsewhere.

All these developments in the use of physical remedies have taken place within the last twelve or eighteen months. I do not at all wish to use language of exaggeration, but the experience that has now been gained entitles us to speak with some confidence. Those who, like myself, have seen the results already obtained, will agree with me that it has now been proven that physical remedies, and especially remedial baths, are of the greatest value in the restoration of disabled soldiers, and also that in many instances the issue as between recovery and permanent crippling often entirely depends upon the scientific use of these remedies. With their timely aid many thousands of men will become again useful citizens; without it they would be condemned to a life-long incapacity. And these things being so, I take it that we are in duty bound to place the issue plainly before the medical profession and the public.

Are we not also bound to take a rather long-sighted view of this matter? The welfare of large numbers of our fellow-citizens during a long future is at stake. I am certain that we shall all agree that the benefit of these remedies cannot be withheld from those who, on account of their disabilities, have now been discharged from the Army. The case of the ex-soldiers is in many ways more urgent than that of the soldiers for whom these new orthopædic installations have now been provided. The discharged man drifts, half-cured, through the hospitals to his own home, and he no longer has the advantage of prolonged and patient restorative treatment. It is true that he has his pension. He has at present but little besides. A few weeks ago the Minister of Pensions informed the House of Commons that there were more than 140,000 discharged men under his Ministry, and still the numbers grow. The restoration of these men to active life has yet to be accomplished. I have lately seen some of the great institutions for the re-education of disabled soldiers, which, under the French and Belgian Governments, are restoring in hundreds and thousands, to some degree of activity and fitness, these shattered members of society. The men are grouped in great centres, where every influence, both physical and mental, is favourable to recovery. In these institutes physical remedies and physical treatment are inseparably combined with industrial or agricultural training, and they are placed—as they ought to be placed—under skilled direction, and with an accurate system of measurements and records.

In order to deal adequately or wisely with the restoration of the wounded we should realise that it is a very large and complicated issue, in which medical, industrial, social, and national problems are included. Surely this is a field for the ablest thinkers and the ablest organisers. Many elements and many methods must be employed. It is impossible to suppose that one method or one prescription alone—such as curative workshops or remedial exercises—can meet the needs of these disabled thousands. Each man must be considered as an individual, and individually dealt with. Not only the function of the limb, but often the mental and moral nature of the sufferer, must be cared for and, as far as possible, restored. I would only plead here that no system of re-education of the discharged soldier can be complete or adequate unless physical remedies under proper direction are included in its programme.

I have now briefly indicated the work that lies before us in regard to physical remedies. But where are the experts who are competent to direct special treatment of this kind? They are needed in the health resorts, in hospitals and clinics, and last, but not least, they will be needed in the great centres of re-education. We hear a great deal about the shortage of doctors, but how many medical men are there who are specialists in physical treatment? They say it takes four years to make a bookbinder. Happily, in this war our people have shown a marvellous adaptability to new and unexpected

☞ The previous article appeared on August 11, p. 379.

demands, but hydrologists cannot be made in a day. Knowledge, experience, technique, and practical skill must be patiently acquired in this as in every other special department of medicine.

Unfortunately, in our country there is no provision for instruction in this branch of science. In all our universities and colleges it is conspicuous by its absence. At a time when the most thoughtful and cultivated people in this country, and in all countries, are more and more resorting to physical remedies by means of climates and waters and baths, we have been content to let the health-seekers go abroad. We have gone abroad for experts and specialists to advise our invalids in all these matters. If we wish to investigate physical methods, we have ourselves to go abroad and sit at the feet of foreign professors for the necessary foundation of experimental science and research. As a consequence of this neglect, when we are driven to employ the same methods for our soldiers, is it wonderful that we are at first rather lacking in skill, experience, and technique?

Shortly before the outbreak of war a memorial was presented to the Senate of the University of London praying for the establishment of a Chair of Medical Hydrology and Climatology in London. This memorial, which bore the signature of representative members of the medical profession, set forth that these subjects taken together formed a branch of medicine of such importance that it ought to be systematically taught. It showed that the lack of proper teaching in medical subjects had been the source of many evils, opening the door for irregular and empirical practice; that from the early days of medicine these modes of treatment had been widely employed, and had survived every change of medical opinion, and that systematic teaching may be expected to do much to counteract ignorant and unscientific methods in this branch of practice; that both climatic and hydrological treatment of disease were now more widely valued and practised in all countries than at any former period; that in the British Islands they were in the unfortunate position of being practised without being taught. If any art is worthy to be practised it was necessary in the public interest that it should be taught. The memorialists further set forth that very considerable provision for such teaching had been set up in Continental countries; that in France, for example, a Chair of Hydrology had been founded in 1891, and an Institute of Hydrology had been added to the Collège de France in 1913; that in Germany there were two professorships, as well as regular lectures in ten other universities; and that there were one or more university professors in charge of these subjects in Austria-Hungary, Italy, and Switzerland, besides lectureships in Holland, Sweden, and Belgium. It was therefore proposed that teaching and laboratory facilities should be made available in London:—

1. For senior students in the London medical schools. 2. For practitioners in London who are interested in (a) hydrotherapy; (b) medicinal springs and climates. 3. For medical men who come to London from the country or more distant parts of the Empire, for the express purpose of special study in these branches of therapeutics. 4. Also, and more particularly, for junior graduates who propose to follow the practice of medicine at British health resorts—whether with waters and baths or at climatic stations at home or in the Colonies. 5. Lastly, for research work in various directions; for example, into the effects of heat and cold in different media, and into the chemical and physical properties of water.

The practitioner who devoted himself to the use of waters and climates might be regarded as a specialist, as respects the remedies that he employed. In this country alone was he at present without the means of special instruction and research. Both for the credit of the practitioner and in the public interest, it would be well that this want should be supplied.

Should the University of London set up this professorship, not only would the theoretical study of medical climatology and of hydrology be widely extended, but also light would incidentally be shed upon the singularly rich endowment of the British Empire in health resorts. In India, South Africa, Australia, Canada, New Zealand, there are large numbers both of waters and climates of the first importance awaiting scientific study.

Like many other projects, this proposal to fill a gap in University and medical teaching has been held up by the war. Now, however, after three years, we are more than ever disposed to make good our national deficiencies in educational questions. Here in Cardiff a great educational question is being threshed out. By the kindness of Colonel Bruce Vaughan I have had the opportunity of reading some of the evidence that was laid before the Royal Commission on University Education in Wales. It is very interesting to see how greatly in these days a sense of the value of science has permeated the public mind, and that the case for University education is now to a large extent based upon research. References to the pre-eminent value of research are freely scattered through the evidence. The Council of the University College of South Wales and Monmouthshire, according to their Charter, desire to "make provision for research, and for the advancement and dissemination of knowledge." Those who advocate the foundation of a National School of Medicine in Wales say the same thing. The educational and the research work must go hand in hand. In the words of Colonel Bruce Vaughan, "the whole thing hinges on research," and Sir William James Thomas emphasises the importance of "furthering the work of investigation and research."

In speaking to you to-day on the medical or physical side of orthopædic treatment of disabled soldiers, I should entirely fail unless I made it quite clear that the use of physical remedies must have a broad basis of exact science. The art of medicine in all its branches rests on many sciences, and is everywhere ultimately founded upon repeated and painstaking research. I hope that in the new National School of Medicine in Wales, if it is happily realised, provision may be made for teach-

(*Concluded on page 406.*)

**PHYSICAL ORTHOPÆDICS** (*continued from p.* 398.)

ing and research in hydrology and the allied branches of physical treatment; for in these matters the Continental nations have certainly stolen a march on us.

Not many weeks ago there passed away in Vienna a man who to the close of a long life continued to be a great learner and a great teacher in this department of medicine. Professor Wilhelm Winternitz, who is sometimes called "the father of modern hydrotherapy," has, together with his pupils, given us almost all that we possess of precise modern data in the use of heat and cold in remedial baths. Only lately he sent me a study of his old age on the effect of cold in tuberculosis, a matter which, of course, underlies our fresh air and sanatorium treatment of consumption. For many years Winternitz has taught in his University to students drawn from all parts; and I think it must have been pleasant to him to feel that he had had an opportunity of making this great contribution to medicine, and that he was leaving behind him, in every capital of Europe, pupils and disciples who are imbued with his doctrine and will keep alive his spirit.

As another example of a great teacher in this field let me mention Dr. Felix Garrigou, who still happily survives. For nearly thirty years he has been Professor of Hydrology in the University of Toulouse. It has been his custom to give an opening introductory lecture at the beginning of his sessional course. "I have been glad," he says in one of these discourses, "to reserve this first lecture to my colleagues and pupils of the faculty, and to dedicate it to the young generation of future hydrologists, whose mission it will be to walk in the pathways opened for them by the labours of those who have preceded them, and their duty by serious research to maintain in the foremost place the science of hydrology in France."

I cordially wish all success to the new department of King Edward VII.'s Hospital, in which physical remedies will be used for the restoration of wounded soldiers, and later, I hope, for the relief of civilians also; and I couple with that the hope that not alone in Continental cities like Vienna and Toulouse, but in English cities also like London and Cardiff, hydrology may not merely be practised, but studied and taught.

